# Machine Learning-Predicted Length of Hospital Stay in Pediatric Appendicitis: An Exploratory Comparative Study of Clinical and Ultrasound-Based Models with Explainable Artificial Intelligence and Bayesian Analysis

**DOI:** 10.3390/medsci14050556

**Published:** 2026-09-09

**Authors:** Kannan Sridharan, Gowri Sivaramakrishnan

**Affiliations:** 1 Department of Pharmacology & Therapeutics, College of Medicine & Health Sciences, Arabian Gulf University, Manama P.O. Box 26671, Bahrain; 2 Bahrain Defence Force Royal Medical Services, Riffa P.O. Box 28743, Bahrain

**Keywords:** appendicitis, machine learning, artificial intelligence, Bayesian predictions

## Abstract

**Background:** Accurate prediction of hospital length of stay (LOS) in pediatric appendicitis is essential for optimizing resource allocation, guiding discharge planning, and improving patient outcomes. This study aimed to explore the potential of machine learning (ML) models for predicting LOS using clinical and ultrasound features, compare the added value of ultrasound findings, and provide interpretable insights using explainable artificial intelligence (XAI) and Bayesian analysis. **Methods:** A retrospective cohort of 323 pediatric patients with uncomplicated appendicitis was analyzed from publicly available open-access data from the UCI Machine Learning Repository. Clinical-only and clinical-plus-ultrasound feature sets were used to train Random Forest, XGBoost, and Bayesian Additive Regression Trees (BART) models. Performance was evaluated using mean absolute error (MAE), root mean square error (RMSE), mean absolute percentage error (MAPE), and P20 (percentage of predictions within 20% of actual values) with 95% confidence intervals (CIs) from bootstrap resampling. XAI analysis was employed for model interpretability, with exploratory, hypothesis-generating subgroup analyses and sensitivity analyses conducted across patient demographics and imputation strategies. **Results:** The Random Forest clinical model achieved the best performance (RMSE: 1.66 days, 95% CI: 1.20–2.15; MAE: 1.08 days, 95% CI: 0.80–1.42; P20: 58.7%, 95% CI: 46.0–69.8), significantly outperforming XGBoost (*p* = 0.03 for RMSE). BART demonstrated comparable accuracy (RMSE: 1.57 days, 95% CI: 1.21–2.12). SHAP analysis identified absence of peritonitis and appendix diameter as the most influential predictors. Subgroup analysis revealed a potentially better performance in female patients (RMSE: 1.21 days, P20: 75.8%) but poor accuracy for prolonged LOS ≥6 days (RMSE: 4.13 days, P20: 0%). Sensitivity analysis confirmed robustness of imputation (Spearman’s rho: 0.782–0.927). **Conclusions:** ML models, particularly Random Forest, predict LOS in pediatric appendicitis using clinical features. XAI provides clinically interpretable insights.

## 1. Introduction

Pediatric appendicitis remains one of the most common surgical emergencies in children, with a lifetime risk of approximately 7–8% and substantial implications for healthcare resource utilization and patient outcomes, and a steady increase in the annual incidence is observed across hospitals in the United States [1]. Length of hospital stay serves as a critical metric for evaluating the quality and efficiency of care delivery, reflecting not only clinical recovery but also influencing healthcare costs, bed management, and patient and family satisfaction [2,3]. Accurate prediction of hospitalization duration can facilitate optimal discharge planning, enhance resource allocation, and inform clinical decision-making regarding the need for surgical intervention versus conservative management, yet traditional approaches to predicting length of stay have relied heavily on clinician intuition and subjective assessments that are often imprecise and inconsistently applied [4]. The emergence of machine learning (ML) methodologies offers a promising alternative, leveraging the ability to analyze complex interactions among multiple clinical variables and identify patterns that may not be readily apparent through conventional statistical approaches [5]. However, the limited interpretability of many ML models has posed a significant barrier to their clinical adoption, as clinicians and healthcare administrators require transparent, explainable tools that can be trusted and effectively integrated into routine practice [6]. A recent systematic review evaluating machine learning models for acute appendicitis concluded that existing research has predominantly focused on diagnostic accuracy, with a conspicuous absence of studies investigating artificial intelligence for predicting length of hospital stay or other clinically actionable outcomes [7]. Furthermore, while ultrasound is commonly employed in the evaluation of pediatric appendicitis to confirm diagnosis and assess disease severity, the incremental value of ultrasound findings beyond clinical parameters for predicting length of stay remains unclear, with limited evidence available to guide clinical practice. The application of explainable artificial intelligence (XAI) techniques coupled with Bayesian methods offers the potential to enhance both predictive accuracy and model interpretability, addressing the critical need for clinically actionable tools in pediatric surgical care. Therefore, this study aimed to explore ML models for predicting length of hospital stay in pediatric appendicitis using clinical and ultrasound features, evaluate the added value of ultrasound findings, and provide clinically interpretable insights through XAI and subgroup evaluations to inform clinical decision-making, discharge planning, and healthcare resource management. The primary outcome of this study is the prediction of hospital length of stay. The secondary outcomes include: (1) the comparative evaluation of predictive performance between clinical-only and clinical-plus-ultrasound feature sets to assess the incremental value of ultrasound findings; (2) the identification and ranking of influential predictors through feature importance analysis and XAI-based explainability; and (3) the exploratory subgroup analyses examining model performance across different patient demographics, management strategies, and length of stay categories.

## 2. Methods

### 2.1. Study Design and Ethics

This retrospective cohort study was conducted at Children’s Hospital St. Hedwig in Regensburg, Germany, utilizing data from pediatric patients admitted with abdominal pain and suspected appendicitis between 2016 and 2021 [8,9]. The study was approved by the Ethics Committee of the University of Regensburg under approval numbers 18-1063-101, 18-1063_1-101, and 18-1063_2-101, and was performed in accordance with applicable guidelines and regulations.

### 2.2. Study Procedure

A stepwise protocol for easy understanding of study methods is as follows. The overall prediction workflow comprised four sequential phases: (1) data collection and preprocessing, (2) feature set construction, (3) model training and hyperparameter tuning, and (4) model evaluation and interpretation. First, all clinical and ultrasound data were extracted from electronic health records at the time of admission, prior to any surgical intervention or definitive treatment decisions. Second, two distinct feature sets were constructed: a clinical-only set comprising 23 demographic, symptomatic, physical examination, and laboratory variables; and a clinical-plus-ultrasound set incorporating the same clinical variables along with prespecified ultrasound parameters. Third, the dataset was randomly split into training (80%) and testing (20%) sets using stratified sampling based on length of stay categories to preserve outcome distribution and machine learning models were evaluated.

Patients diagnosed with uncomplicated appendicitis were included in the present study. Patients with complicated appendicitis, perforation, or abscess formation were excluded to ensure a homogeneous cohort, as these conditions introduce substantial clinical heterogeneity (such as surgical complexity, postoperative complications, and variable recovery trajectories) that would confound length of stay predictions and limit the generalizability of findings to the more common uncomplicated disease presentation. For each patient in the original study, comprehensive clinical data were collected at the time of admission, including demographic information such as age, sex, height, weight, body mass index (BMI), along with length of hospital stay. Laboratory parameters included white blood cell count, segmented neutrophils, neutrophil percentage, and C-reactive protein, among others. Physical examination findings included the presence of migratory pain, right lower quadrant pain, contralateral rebound tenderness, coughing pain, nausea, loss of appetite, and body temperature measurements. Clinical scoring systems were applied to each patient, with the Alvarado Score and Pediatric Appendicitis Score calculated to aid in diagnostic assessment [10,11]. Abdominal B-mode ultrasound examinations were performed for most patients, with variable number of views and extracted detailed findings including appendix diameter, appendix visualization status, presence of free fluids, appendicolith, wall layer integrity, target sign, perfusion, perforation, surrounding tissue reaction, appendicular abscess, and pathological lymph nodes. The final diagnosis was histologically confirmed for patients who underwent surgical intervention, while conservatively managed patients were labelled as having appendicitis if they had an Alvarado Score or Pediatric Appendicitis Score of four or greater and an appendix diameter of six millimeters or more. All data were extracted from electronic health records and systematically organized for analysis, with the complete dataset deposited in the UCI Machine Learning Repository under DOI 10.5281/zenodo.7669442, ensuring transparency and reproducibility of the study findings [9]. This manuscript is reported in line with the recommendations from the GAMER statement [12].

### 2.3. Variables Included

We utilized length of hospital stay measured in days as the outcome variable. The predictor set was organized into two distinct model frameworks: a clinical-only model comprising twenty-three variables including patient demographics (age, sex, and BMI), clinical signs and symptoms (migratory pain, lower right abdominal pain, contralateral rebound tenderness, coughing pain, nausea, loss of appetite, peritonitis, psoas sign, and ipsilateral rebound tenderness), laboratory parameters (white blood cell count, neutrophil percentage, hemoglobin, thrombocyte count, red cell distribution width, C-reactive protein (CRP), and the presence of ketones, red blood cells, and white blood cells in urine), and clinical assessment findings (body temperature and stool characteristics); and an enhanced clinical-plus-ultrasound model which incorporated all clinical predictors along with two additional ultrasound parameters, namely appendix diameter and the presence of free fluids. The following variables were categorized as binary (yes/no): ketones, red blood cells, and white blood cells in urine. The scores were deliberately excluded from the predictor set to avoid redundancy and multicollinearity, as they are a composite measure derived from several individual clinical signs and symptoms already included in our models, including migratory pain, nausea, loss of appetite, coughing pain, rebound tenderness, and a few laboratory parameters, and their inclusion would have obscured the independent contributions of these component variables to length of stay prediction. All categorical predictors were converted to factor variables and subsequently one-hot encoded for machine learning implementation, while continuous variables were analyzed in their original numeric form without transformation. Ultrasound images were not available in the original dataset, which constrained image-based analysis.

The cohort comprised 323 patients with uncomplicated appendicitis, with a mean age of 11.33 ± 3.29 years and a slight male predominance (58.8%). The mean length of hospital stay was 3.97 ± 1.56 days, ranging from 1 to 11 days, indicating a generally short hospitalization period consistent with uncomplicated appendicitis management. Laboratory parameters showed expected findings, with mean WBC count of 13.33 ± 4.77 × 10^3^ cells/mm^3^, neutrophil percentage of 74.45 ± 13.12%, and CRP level of 24.87 ± 40.28 mg/dL, reflecting the inflammatory response associated with appendicitis. Among categorical variables, the most prevalent clinical features were lower right abdominal pain (97.8%), nausea (59.3%), loss of appetite (49.8%), and free fluids on ultrasound (46.4%), while generalized peritonitis was relatively uncommon (4.3%).

### 2.4. Statistical Analysis

Initial data preparation involved inspection of variable distributions, handling of column naming inconsistencies, and conversion of categorical variables to factors. Normality of the data was assessed using Kolmogorov–Smirnov test. Missing data were assessed using the naniar package, and multiple imputations were performed using the mice package with predictive mean matching, generating five imputed datasets with 20 iterations each. A complete case analysis was also conducted as a sensitivity analysis to evaluate the robustness of the imputation strategy. Multicollinearity among predictors was evaluated using variance inflation factor (VIF) analysis for numeric variables, with a threshold of 5 indicating moderate concern and 10 indicating high concern, while Cramér’s V was calculated for categorical associations, along with correlation coefficient (r > 0.7) [13]. Only hemoglobin and RBC count had high correlation (r = 0.74) and hemoglobin was retained for further analysis. One-hot encoding was applied to all categorical predictors using the recipes package, dropping the first category to avoid dummy variable trap, and the dataset was split into training (80%) and testing (20%) sets using stratified sampling based on length of stay categories (≤3 days, 4–5 days, ≥6 days) to preserve outcome distribution [14]. Random seeds were set at multiple stages (42, 123, 456, 789) to ensure complete reproducibility of all random processes.

Two ML algorithms were implemented for predictive modeling: Random Forest using the ranger package and XGBoost, each trained on both clinical-only and clinical-plus-ultrasound feature sets. Random Forest and XGBoost were selected as the primary machine learning algorithms due to their proven ability to handle non-linear relationships, high-dimensional data, and complex interactions among predictors without requiring stringent distributional assumptions, which are common limitations of traditional linear regression models [15,16]. Ensemble tree-based methods are particularly suited for clinical prediction tasks as they inherently accommodate mixed data types (continuous and categorical variables), are robust to multicollinearity, and provide built-in feature importance rankings. Bayesian Additive Regression Trees were additionally employed to offer a probabilistic framework that complements frequentist approaches, enabling uncertainty quantification through posterior distributions [17]. Hyperparameter tuning was performed using 5-fold cross-validation with randomized search of 100 iterations, optimizing for root mean square error (RMSE). Final models were trained on the full training set with best-performing hyperparameters, and predictions were generated on the held-out test set. Model performance was evaluated using multiple metrics including mean absolute error (MAE), RMSE, mean absolute percentage error (MAPE), and the P20 metric representing the percentage of predictions within 20% of actual values. The performance metrics reported represent estimates that may be subject to optimistic bias due to the imputation-before-split approach. Bootstrap resampling with 1000 iterations was employed to compute 95% confidence intervals (CI) for all performance metrics. Feature importance was evaluated for the best performing ML model, Random Forest, using permutation importance approach.

Bayesian Additive Regression Trees (BART) were implemented using the dbarts package with 50 trees, 1000 posterior draws, and 500 burn-in iterations, generating predictions and partial dependence plots for the top five to ten important variables. SHAP (SHapley Additive exPlanations) analysis was performed on the best-performing Random Forest models using the fastshap package to provide global and local interpretability, including beeswarm plots, mean absolute SHAP bar plots, dependence plots for top features, and heatmaps of SHAP values. SHAP values for variables were also compared between the predicted cases within 20% of actual values (P20 success) and outside the 20% of actual values (P20 failure) groups.

Additional exploratory hypothesis-generating subgroup analyses were conducted for age groups (<10 years, 10–14 years, ≥15 years), sex, management strategy (conservative versus primary surgical), BMI categories (underweight: <18.5 kg/m^2^; normal: 18.5–24.9 kg/m^2^; overweight/obese: 25 kg/m^2^), and length of stays (long: ≥6 days; medium: 4–5 days; and short: ≤3 days), with performance metrics recalculated within each subgroup and compared using bootstrap methods.

A comprehensive sensitivity analysis was conducted using complete case analysis with listwise deletion to compare against imputed results, assessing consistency of performance metrics and feature importance rankings using Spearman correlation for the Random Forest model predictions. This sensitivity analysis had all preprocessing steps performed within the training data and subsequently applied to the test set, and the results confirmed the stability of our primary findings. All statistical tests were two-sided, with *p*-values adjusted for multiple comparisons using Bonferroni correction where appropriate. Statistical significance was set at *p* ≤ 0.05 after adjustment. All analyses were carried out using R (4.5.2).

## 3. Results

### 3.1. Demographics of Included Patients

The missing data pattern was predominantly confined to urine parameters (ketones, RBC, and WBC in urine), each with approximately 26% missingness, likely due to these tests not being routinely ordered in all patients with suspected appendicitis (Electronic Supplementary Table S1). Other variables exhibited substantially lower missing proportions, ranging from 0.31% to 9.6%, with appendix diameter missing in 9.6% of cases, primarily attributable to technical limitations in ultrasound visualization. Given that missingness was not concentrated in the outcome variable (length of stay had 0% missing) and appeared largely random across the cohort, multiple imputation using predictive mean matching was deemed appropriate to preserve statistical power and minimize bias. The imputation was successful for all the missing variables and a summary of the demographic characteristics of the study population across the original, imputed, training, and testing datasets are presented in Table 1, demonstrating overall consistency and successful data splitting for model development and validation. The imputed dataset maintained distributions nearly identical to the original data, indicating that the multiple imputation procedure effectively preserved the underlying data structure without introducing substantial bias.

### 3.2. Machine Learning Analysis

Comparison between the training (n = 260) and testing (n = 63) cohorts revealed no statistically significant differences across all variables, confirming successful stratified randomization and ensuring that the testing set adequately represents the overall study population for unbiased model evaluation (Table 1).

The Random Forest model outperformed XGBoost across all performance metrics, demonstrating superior predictive accuracy for length of hospital stay (Table 2). The Random Forest clinical model achieved the lowest RMSE of 1.66 days (95% CI: 1.20, 2.15) and MAE of 1.08 days (95% CI: 0.80, 1.42), correctly predicting length of stay within 20% of actual values for 58.7% of patients (95% CI: 46.0, 69.8). Statistical comparisons revealed that the addition of ultrasound findings did not significantly improve model performance for either Random Forest (*p*-value for RMSE = 0.106 and MAE = 0.604) or XGBoost (*p*-value for RMSE = 0.616 and MAE = 0.730), indicating that clinical predictors alone provide sufficient information for accurate length of stay prediction. However, Random Forest demonstrated statistically significant superiority over XGBoost in both clinical (*p*-value for RMSE = 0.03 and MAE = 0.002) and clinical-plus-ultrasound (*p*-value for RMSE = 0.042 and MAE = 0.006) models, confirming Random Forest as the optimal algorithm for this prediction task.

### 3.3. Features of Importance Analysis

**Figure 1:**
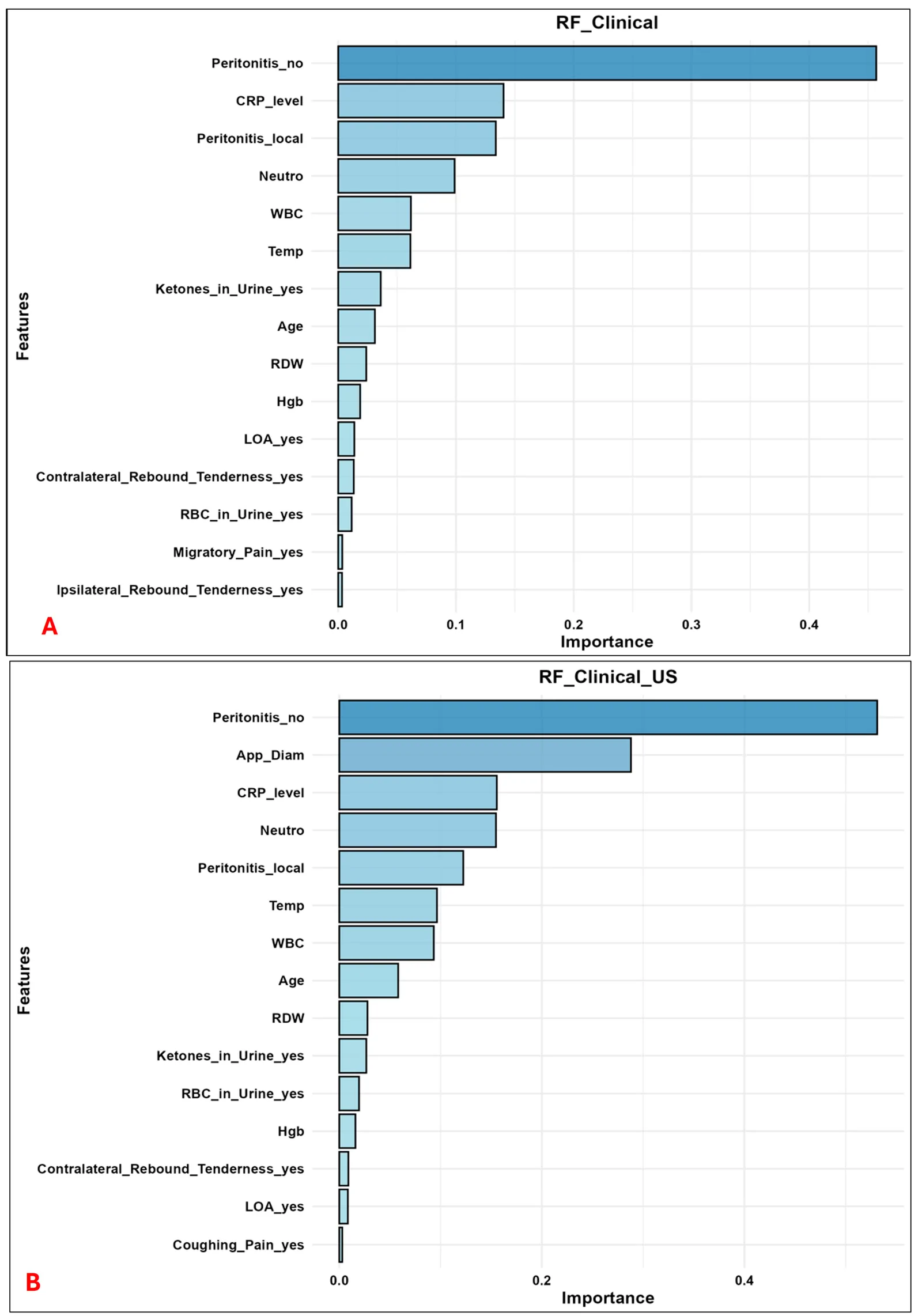
Feature Importance from Random Forest Models. Panel (**A**) displays the top 15 features from the clinical-only model, while Panel (**B**) presents feature importance for the clinical-plus-ultrasound model, ranked by permutation-based importance scores normalized to the most important feature. CRP: C-reactive protein; Hgb: Hemoglobin; LOA: Loss of appetite; Neutro: Neutrophil percentage; RBC: Red blood cell; RDW: Red cell distribution width; Temp: Body temperature; WBC: White blood cell count; App_Diam: Appendix diameter.

The feature importance analysis from the best performing Random Forest revealed that the absence of peritonitis was the most influential predictor in both models, with substantially higher importance scores than all other variables (Figure 1). In the clinical-plus-ultrasound model, appendix diameter emerged as the second most important feature, while other clinical parameters including CRP, neutrophil percentage, WBC count, body temperature, and the presence of ketones in urine demonstrated moderate and relatively similar importance contributions, suggesting that a combination of inflammatory markers and clinical signs collectively drives length of stay predictions in pediatric appendicitis.

### 3.4. BART Analysis

The BART models (Table 3) demonstrated performance comparable to the Random Forest algorithm, with the clinical model achieving an RMSE of 1.57 days (95% CI: 1.21, 2.12) and MAE of 1.07 days (95% CI: 0.87, 1.50), which were nearly identical to the best-performing Random Forest clinical model (RMSE: 1.66 days, MAE: 1.08 days). Like the ML models, the addition of ultrasound findings to BART was observed with modest changes in the performance metrics.

**Figure 2:**
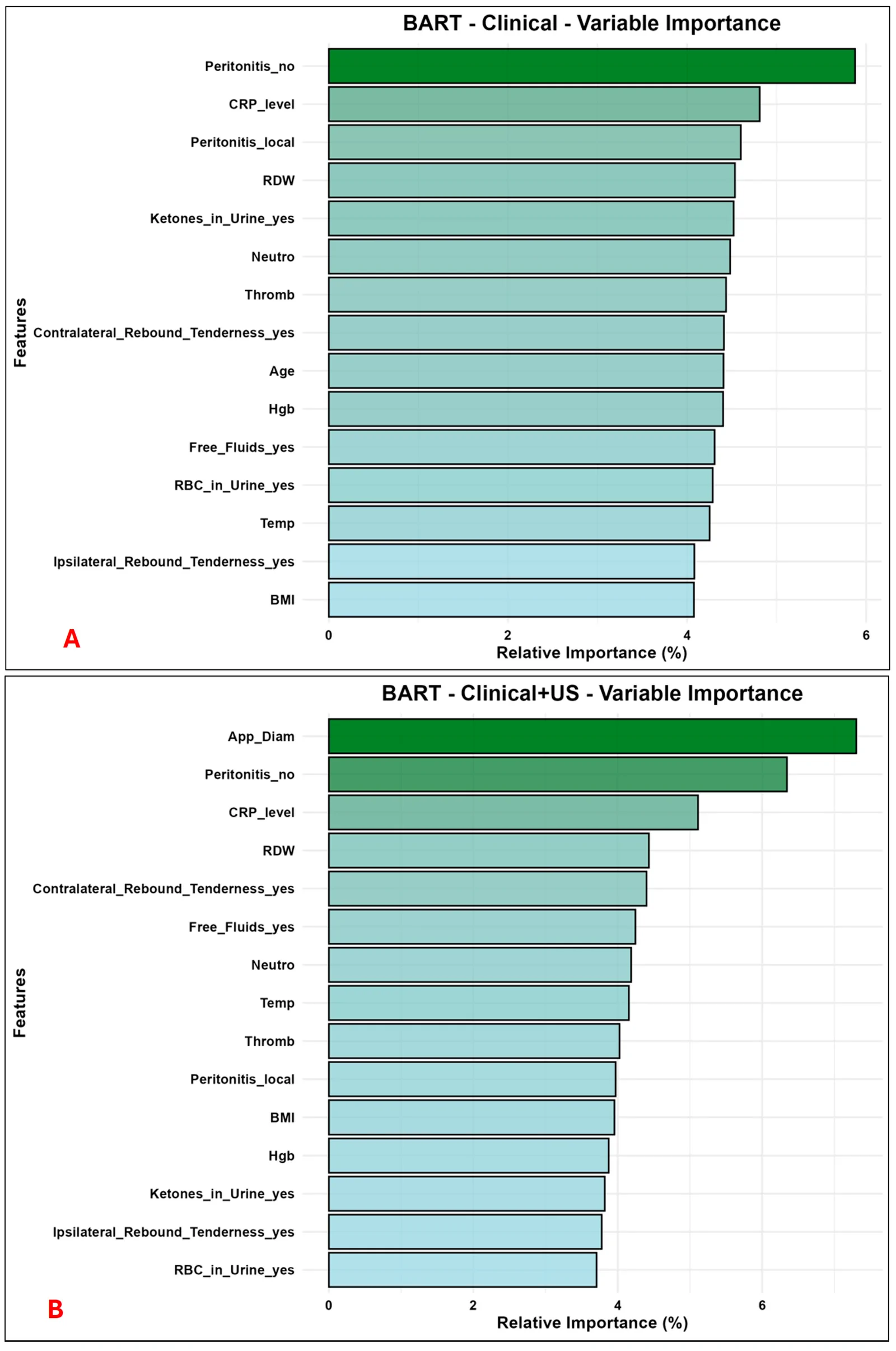
Feature Importance from BART Models. Panel (**A**) displays the variable importance for the clinical-only model, while Panel (**B**) presents feature importance for the clinical-plus-ultrasound model, expressed as percentage contributions normalized to sum to 100%. CRP: C-reactive protein; Hgb: Hemoglobin; Neutro: Neutrophil percentage; RBC: Red blood cell; RDW: Red cell distribution width; Temp: Body temperature; Thromb: Thrombocyte count; WBC: White blood cell count; BMI: Body mass index; App_Diam: Appendix diameter.

The BART variable importance analysis identified the absence of peritonitis as the predominant predictor in both models, consistent with the Random Forest findings, although the importance scores were more evenly distributed across features compared to the Random Forest models where peritonitis dominated substantially (Figure 2). In the BART clinical-plus-ultrasound model, all features demonstrated relatively similar importance contributions ranging from 6.5% to 9.7%, with appendix diameter, C-reactive protein, and peritonitis localization emerging as the top contributors, suggesting that BART captures more balanced additive effects from multiple predictors rather than relying heavily on a single dominant feature.

**Figure 3:**
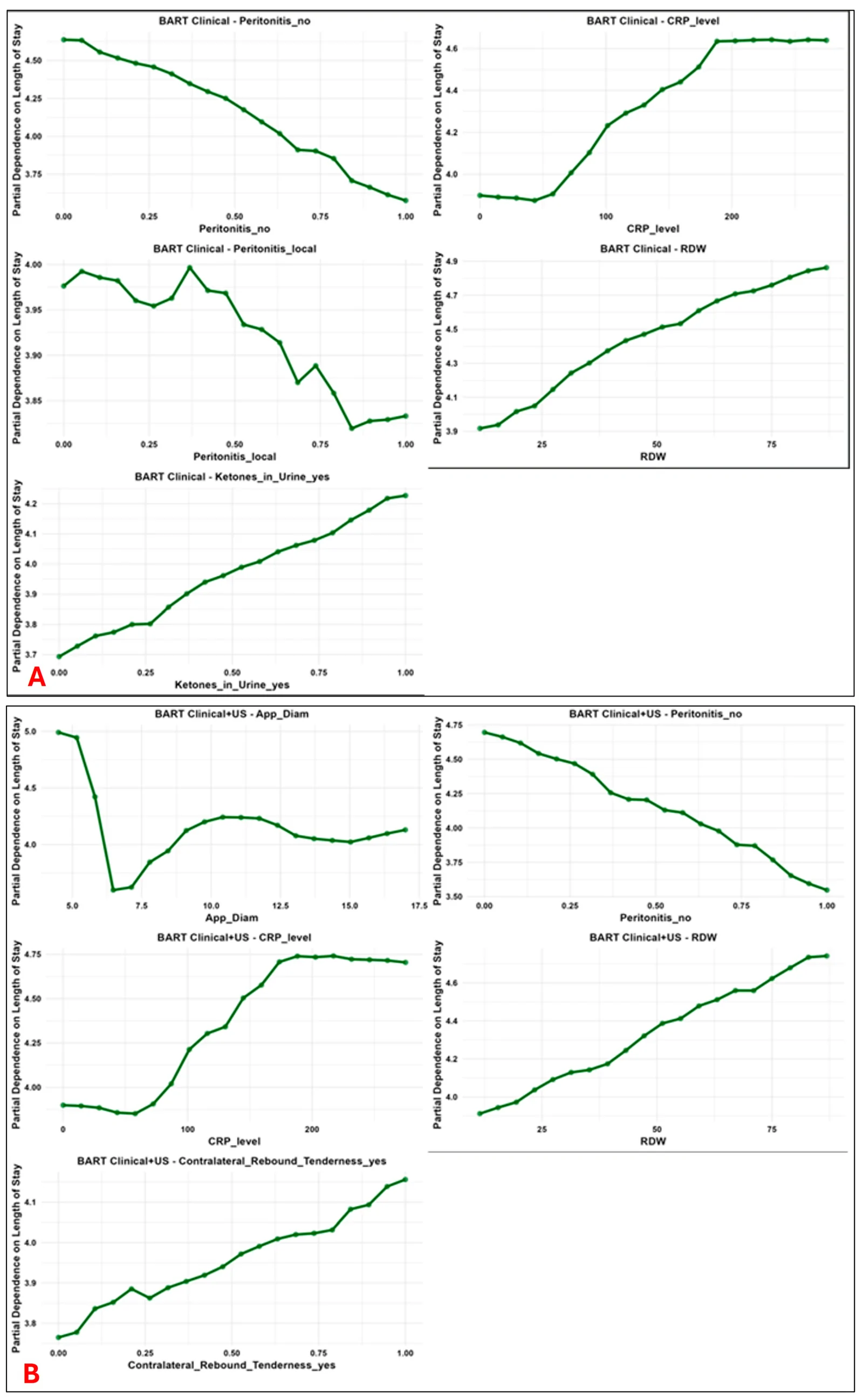
Partial Dependence Plots for BART Models Predicting Length of Hospital Stay. PDP illustrating the marginal effects of the top predictor variables on the predicted hospital length of stay in pediatric appendicitis patients with Panel (**A**) related to Clinical model, and Panel (**B**) is Clinical + US model. CRP: C-reactive protein; RDW: Red cell distribution width; App_Diam: Appendix diameter.

The PDP demonstrates how the predicted hospital length of stay varies across different ranges of the top clinical and ultrasound features within the BART models (Figure 3). Across both the clinical (Panel A) and combined clinical + US (Panel B) models, higher CRP levels, greater RDW, and the presence of specific clinical signs generally correlate with an increased predicted length of stay. Conversely, the absence of peritonitis strongly predicts a shorter hospital stay in both models.

### 3.5. SHAP Analysis

**Figure 4:**
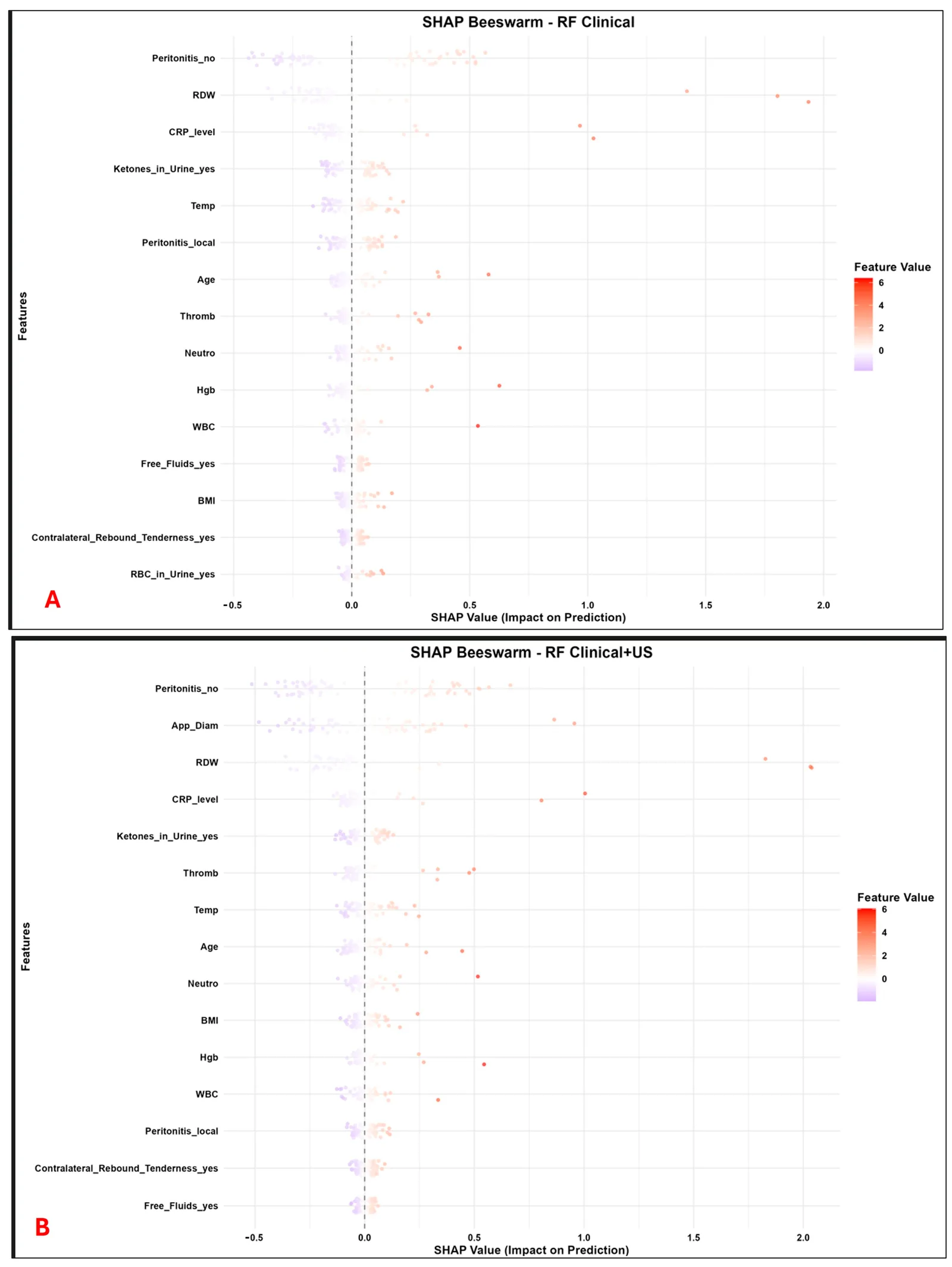
SHAP Beeswarm Plots for Random Forest Models. Panel (**A**) displays the SHAP summary plot for the clinical-only model, while Panel (**B**) presents the beeswarm plot for the clinical-plus-ultrasound model, with features ranked by mean absolute SHAP values and colored by feature value (red indicating high values, blue indicating low values). CRP: C-reactive protein; Hgb: Hemoglobin; Neutro: Neutrophil percentage; RBC: Red blood cell; RDW: Red cell distribution width; Temp: Body temperature; Thromb: Thrombocyte count; WBC: White blood cell count; BMI: Body mass index; App_Diam: Appendix diameter.

The SHAP analysis identified the absence of peritonitis as the most influential predictor in both models, with high feature values consistently associated with shorter length of stay, as evidenced by negative SHAP contributions (Figure 4). In the clinical-plus-ultrasound model, appendix diameter emerged as the second most important predictor, showing that larger appendiceal diameter was associated with prolonged hospitalization. The beeswarm plots revealed that inflammatory markers including CRP, WBC count, and neutrophil percentage demonstrated bidirectional effects, with elevated values generally contributing to longer length of stay, while the presence of ketones in urine and body temperature showed more heterogeneous patterns across patients.

The SHAP-based P20 subgroup analysis revealed that patients with predictions falling outside the 20% accuracy threshold exhibited higher mean absolute SHAP values for age and white blood cell count in the clinical model, and for hemoglobin, thrombocyte count, and appendix diameter in the clinical-plus-ultrasound model, suggesting these features contributed to less accurate predictions (Electronic Supplementary Table S2). However, after Bonferroni correction for multiple comparisons, none of the observed differences in SHAP values between the P20-success and P20-failure groups reached statistical significance (all adjusted *p*-values > 0.05), indicating that the model’s prediction errors were not systematically driven by any single predictor.

### 3.6. Exploratory Subgroup Analysis

The exploratory subgroup analysis revealed variations in model performance across different patient subgroups, with potentially better predictive accuracies observed in female patients and those in the medium length of stay category of 4–5 days, while the poorest performance was associated in patients with prolonged hospitalization of 6 or more days (Table 4). Across all subgroups, the addition of ultrasound findings was observed with modest changes in the performance metrics.

### 3.7. Sensitivity Analysis

**Figure 5:**
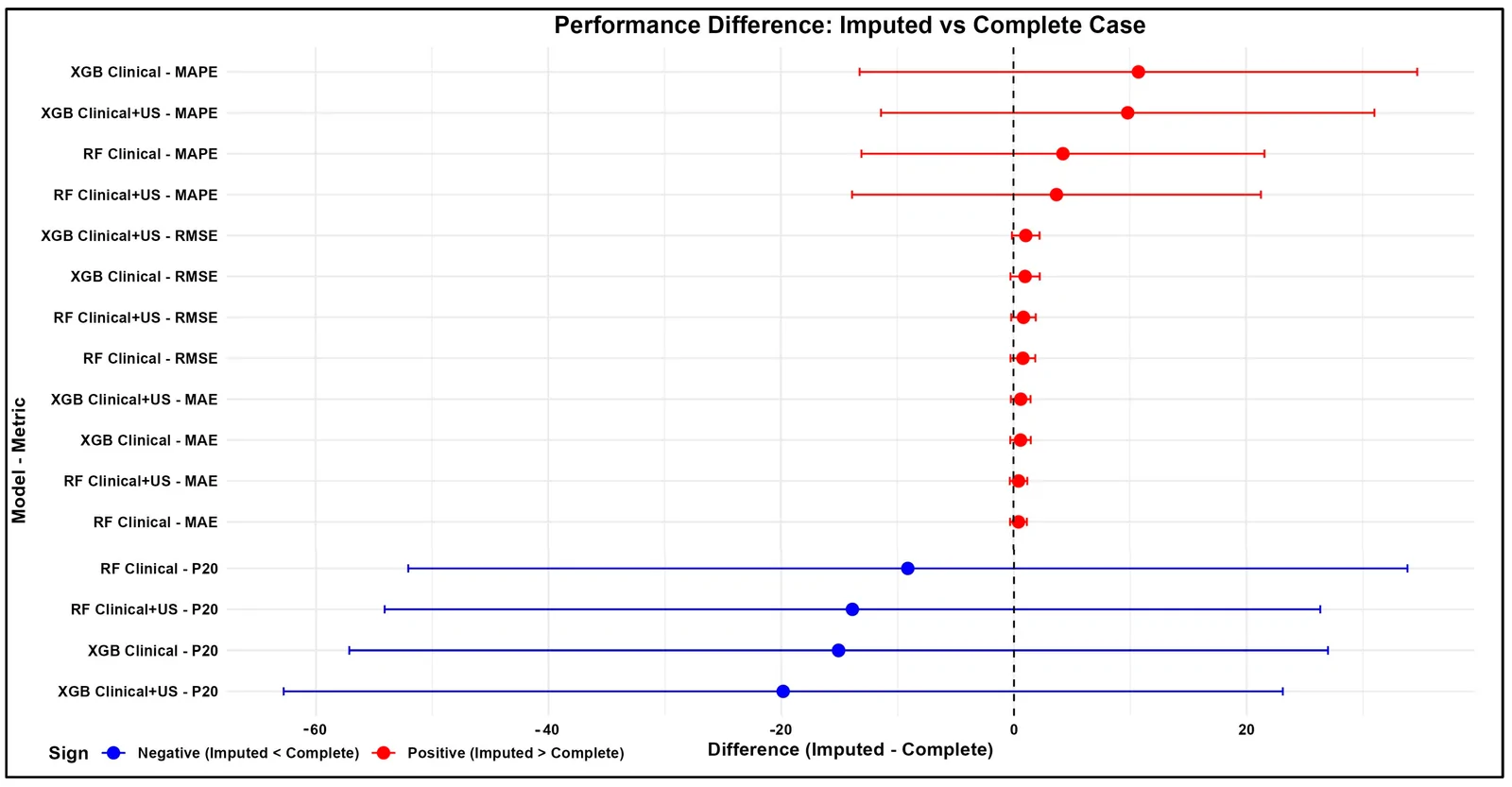
Sensitivity Analysis Comparing Random Forest Performance Between Imputed and Complete Case Datasets. The forest plot displays the absolute performance difference (Imputed minus Complete Case) across various model architectures (XGB: XGBoost; RF: Random Forest) and feature sets (Clinical vs. Clinical + US). Red markers indicate a positive difference (Imputed > Complete Case), while blue markers indicate a negative difference (Imputed < Complete Case). Central points represent the point estimates of the difference, and horizontal error bars denote the corresponding 95% CI. The vertical dashed line at zero represents the line of identity (no difference in performance). MAPE: Mean Absolute Percentage Error; RMSE: Root Mean Squared Error; MAE: Mean Absolute Error; and P20: Proportion of predictions within 20% of actual values.

The sensitivity analysis comparing imputed and complete case datasets revealed consistently lower RMSE and MAE values across all models in the complete case analysis, suggesting that complete case data yielded more accurate predictions, though CI for the differences between imputed and complete case metrics crossed zero for all comparisons, indicating no statistically significant differences between the two approaches (Figure 5).

**Figure 6:**
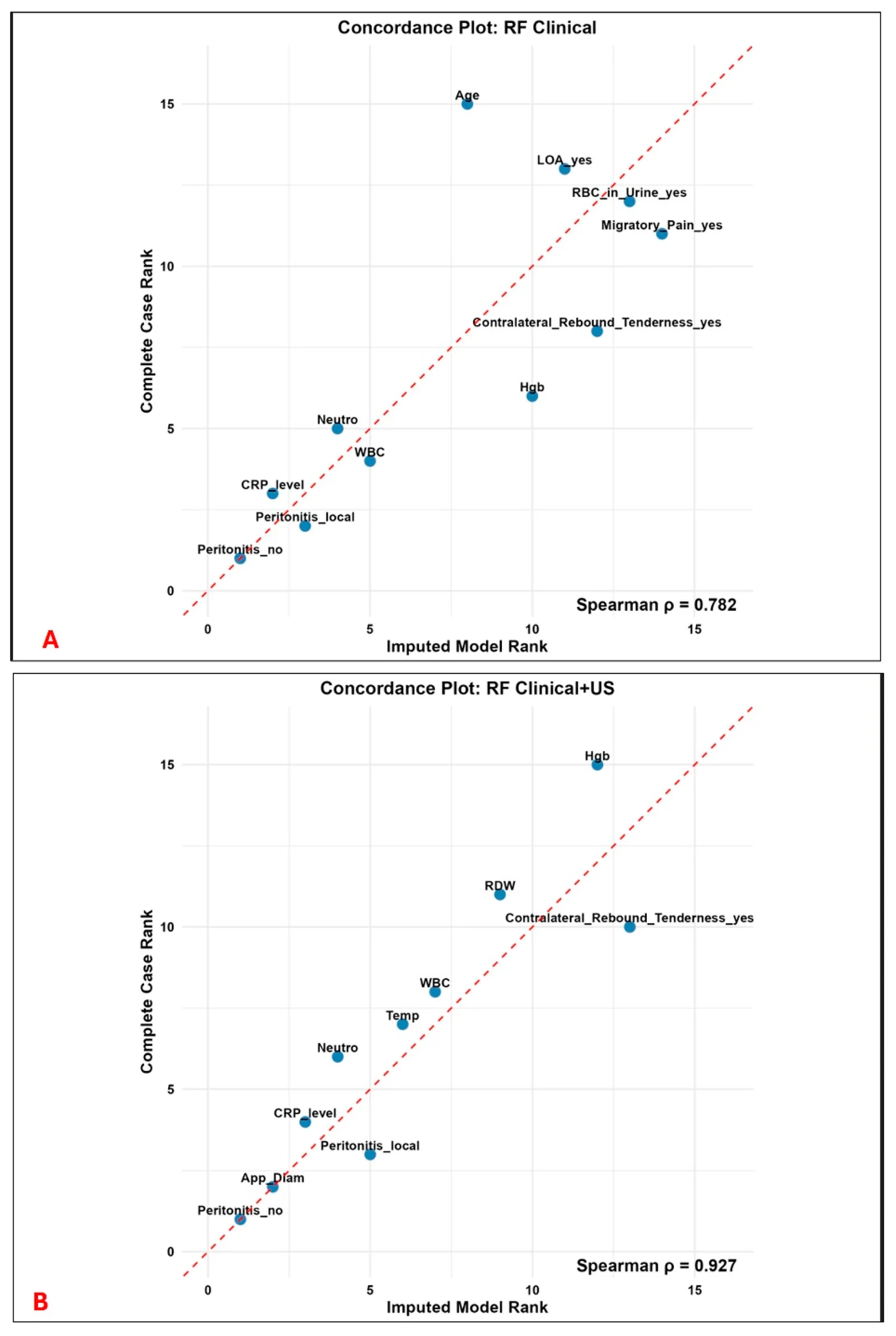
Feature rank concordance analysis for Random Forest models utilizing imputed versus complete case data. Scatter plots illustrates the rank-order consistency of predictive features between the imputed dataset (*x*-axis) and complete case dataset (*y*-axis) related to the Random Forest algorithm for: (**A**) the Clinical feature model (Spearman’s rho = 0.782), and (**B**) the Clinical + Ultrasound (US) feature model (Spearman’s rho = 0.927). The diagonal red dashed line represents perfect rank agreement. CRP: C-reactive protein; Hgb: Hemoglobin; Neutro: Neutrophil percentage; RBC: Red blood cell; RDW: Red cell distribution width; Temp: Body temperature; Thromb: Thrombocyte count; WBC: White blood cell count; BMI: Body mass index; App_Diam: Appendix diameter.

The feature importance ranking revealed strong rank correlation between imputed and complete case datasets, with Spearman correlation coefficients of 0.782 for the Random Forest Clinical model and 0.927 for the Random Forest Clinical + US model (Figure 6). The common features identified across both imputation approaches consistently included peritonitis status, appendix diameter, CRP, neutrophil percentage, and hemoglobin.

## 4. Discussion

### 4.1. Statement of Key Findings

The key findings of this exploratory study demonstrate that machine learning models, particularly Random Forest, can predict length of hospital stay in uncomplicated pediatric appendicitis using readily available clinical parameters, achieving a RMSE of 1.66 days and P20 for nearly 60% of patients. The SHAP analysis suggested that the absence of peritonitis was the most influential predictor of shorter hospitalization, followed by inflammatory markers including CRP, neutrophil percentage, and WBC count, with appendix diameter emerging as important predictors only when ultrasound features were included. In the exploratory subgroup analyses, performance appeared to vary across subgroups, with some suggestion of better accuracy in female patients and those with moderate length of stay, while predictions for prolonged hospitalization (≥6 days) remained challenging. The sensitivity analysis confirmed these findings.

### 4.2. Comparison with Existing Literature

Comparison of our findings with existing literature reveals both concordant and discordant results across conventional regression-based studies of length of stay in appendicitis, though it is important to note that our study focused exclusively on uncomplicated pediatric appendicitis, whereas comparator studies predominantly included adult populations with complicated appendicitis. Martinez-Pérez et al. [18], in their study of 160 adult patients with complicated appendicitis, identified postoperative complications and abdominal drain placement as independent predictors of prolonged length of stay, which partially aligns with our finding that peritonitis status was the most influential predictor; however, the substantially higher complication rates and longer hospitalization in their complicated adult cohort (median length of stay 5 days, 75th percentile 7 days) compared to our uncomplicated pediatric population (mean length of stay 3.97 days) likely explain why additional surgical factors such as drain placement featured prominently in their model but were not applicable in our study. Bancke Laverde et al. [19], in their analysis of adult patients, identified higher age, elevated WBC count and CRP, lower hemoglobin, longer time to surgery, and presence of complicated appendicitis as independent risk factors for prolonged hospitalization, findings that are concordant with our feature importance results demonstrating the strong predictive influence of inflammatory markers including CRP, neutrophil percentage, and WBC count; however, our models did not include time to surgery or conversion rates as we focused exclusively on preoperative clinical and ultrasound parameters available at admission, and the inclusion of complicated cases in their cohort may have amplified the importance of surgical factors that are less relevant in uncomplicated pediatric appendicitis. Angela Trunfio et al. [20], utilizing multiple linear regression in 141 patients with mixed appendicitis severity, found that preoperative length of stay and presence of complications significantly influenced total length of stay with an R^2^ of 0.638, which is concordant with our finding that clinical parameters alone provide sufficient predictive information; however, our ML approach achieved a better predictive performance (RMSE of 1.66 days with Random Forest) compared to their regression model, likely due to the ability of ensemble methods to capture complex nonlinear interactions between predictors and the inclusion of a broader range of clinical variables. The discordance in predictor importance between our study and those of Martinez-Pérez and Bancke Laverde may be attributed to fundamental differences in patient populations, as our exclusively uncomplicated pediatric cohort exhibited lower complication rates, shorter mean length of stay, and different pathophysiological determinants of hospitalization duration compared to adult studies with complicated appendicitis, suggesting that prediction models for length of stay must be tailored to specific disease severity and age groups rather than generalized across all appendicitis populations. Our findings align with and extend the recent body of machine learning literature in pediatric appendicitis. Males et al. [21] developed a random forest model to reduce negative appendectomies in a cohort of 551 pediatric patients, achieving a sensitivity of 99.7% and specificity of 17% for appendicitis detection, with the explicit goal of minimizing missed diagnoses at the cost of higher false positives, a clinical trade-off that contrasts with our approach, which prioritized overall predictive accuracy for length of stay. Similarly, Erman et al. [22] employed a comprehensive ML pipeline on 1980 patients to predict five grades of appendicitis severity, reporting a binary perforation prediction accuracy of 76.4% and AUROC of 0.79, which are comparable to our Random Forest model’s performance for severity prediction when ultrasound features were excluded. Kendall et al. [23], reported that ultrasound features improved diagnostic accuracy but were not essential for predicting management or severity, corroborating our finding that clinical parameters alone provide sufficient information for length of stay prediction. The convergence of these studies, identifying C-reactive protein, white blood cell count, symptom duration, and neutrophil percentage as consistently influential predictors, lends further credibility to our feature importance findings. However, unlike these prior works, our study uniquely addresses the prediction of continuous length of stay as a clinically actionable outcome, employs Bayesian Additive Regression Trees alongside frequentist ensemble methods, and provides extensive subgroup analyses identifying specific patient populations (such as prolonged LOS ≥6 days) where current models fall short. While these studies collectively demonstrate the potential of ML for diagnostic and severity prediction in pediatric appendicitis, our work fills a critical gap by focusing on hospitalization duration prediction, a key metric for resource allocation and discharge planning, and highlights the challenges inherent in predicting the tail end of the LOS distribution. Also, our findings are broadly consistent with the recently published exploratory machine learning study by Turki et al. [24], who evaluated ridge regression, random forest regression, and gradient boosting regression for predicting length of stay in a cohort of 152 pediatric patients with both complicated and uncomplicated appendicitis. In that study, the regression framework achieved an MAE of 2.150 days (95% CI: 1.632–2.846), RMSE of 4.329 days (95% CI: 2.290–6.297), and R^2^ of 0.292 (95% CI: 0.213–0.481), indicating modest predictive performance that was superior to null baseline models. Our Random Forest clinical model demonstrated better accuracy, with an MAE of 1.08 days (95% CI: 0.80–1.42) and RMSE of 1.66 days (95% CI: 1.20–2.15), despite being trained exclusively on uncomplicated appendicitis cases. This discrepancy may be attributed to several factors. First, our study employed a more comprehensive set of clinical predictors (23 variables compared with a more limited set in Turki et al. [24]), including detailed physical examination findings, clinical signs, and laboratory parameters, which may have enhanced the models’ ability to capture the nuanced determinants of hospitalization duration. Second, the inclusion of complicated appendicitis cases in the Turki et al. [24] cohort likely introduced substantial heterogeneity in recovery trajectories, postoperative complications, and treatment complexity, all of which contribute to greater variability in length of stay that is difficult to predict using pre-treatment variables alone. This is supported by the substantially longer median LOS in their complicated subgroup (7 days compared with 2 days in uncomplicated cases) and the extreme right-skewed distribution (range 1–42 days), which contributed to higher prediction errors. Third, differences in healthcare systems, discharge practices, and institutional protocols between Saudi Arabia and Germany may have influenced baseline length of stay patterns and the factors driving prolonged hospitalization. Importantly, both studies converge on the finding that ensemble tree-based methods, particularly Random Forest, demonstrate superior performance compared with simpler regression approaches, and both highlight the significant challenges in accurately predicting prolonged hospital stays. The consistency of feature importance findings across studies, with inflammatory markers, symptom duration, and imaging findings consistently emerging as influential predictors, lends further credibility to the clinical relevance of these variables. These comparisons underscore the need for standardized, multi-center datasets with harmonized predictor definitions to enable more robust cross-study comparisons and external validation of machine learning models for pediatric appendicitis outcomes. From a clinical standpoint, the ability to predict hospitalization duration with a RMSE of approximately 1.6 days and correctly classify nearly 60% of patients within 20% of their actual length of stay represents a preliminary step toward data-informed discharge planning, though the current performance metrics fall short of the precision required for definitive clinical decision-making. An RMSE of 1.6 days translates to a prediction error that could meaningfully impact bed management and patient flow at the aggregate level, but at the individual patient level, the 40% of predictions falling outside the 20% accuracy threshold limits the model’s reliability for guiding specific discharge decisions. CRP was identified as one of the key predictors of length of stay in appendicitis in the present study which is in line with the existing studies [25,26]. Liu et al. demonstrated a non-linear association between admission CRP levels and length of stay in a large cohort of 815 pediatric appendicitis patients undergoing laparoscopic appendectomy [26]. They identified a CRP threshold of 34.13 mg/L, above which each 1 mg/L increase was significantly associated with a 0.013-day prolongation of hospitalization. This quantitative relationship provides external validation for our observation that CRP is a moderately influential predictor, though our SHAP analysis suggests that clinical signs such as the absence of peritonitis and ultrasound parameters like appendix diameter often exert stronger and more immediate influences on predicted length of stay at the point of admission. Collectively, these studies underscore the complementary value of both traditional regression and modern machine learning approaches in understanding the determinants of hospitalization duration. To illustrate the clinical utility of SHAP-based interpretability in a time-sensitive setting, consider a 10-year-old child presenting with right lower quadrant pain, an ultrasound showing an appendix diameter of 9 mm, a CRP of 45 mg/dL, and no evidence of peritonitis on examination. The Random Forest model would generate a baseline predicted length of stay, for instance, 3.2 days. SHAP analysis would then decompose this prediction: the absence of peritonitis would contribute the largest negative effect (shortening the predicted stay by approximately 0.5–0.6 days), while the enlarged appendix diameter (9 mm) and elevated CRP would each add roughly 0.3–0.4 days. The net effect would be a final prediction of approximately 3.3–3.5 days. In practice, this allows the clinician to see not only the final estimate but also the directional influence of each feature at the point of care. If the child’s appendix diameter were 12 mm instead of 9 mm, the SHAP contribution from this feature would increase proportionally, lengthening the predicted stay and prompting earlier consideration of enhanced monitoring or proactive discharge planning. Such real-time, feature-level transparency can support shared decision-making with families and help triage patients for early discharge pathways, all within the first hour of admission. The modest P20 of approximately 60% suggests that while these models may offer supportive information to clinicians, they should not replace clinical judgment or serve as the sole basis for discharge planning or resource allocation. The practical implications are therefore most relevant for population-level applications, such as identifying broad trends in anticipated bed occupancy or flagging patients who may require closer monitoring, rather than for precise, patient-specific discharge timing. Future improvements in predictive performance, potentially through the incorporation of dynamic in-hospital data or larger multi-center cohorts, would be necessary before these models can be confidently integrated into routine clinical workflows for individual patient management. The finding that ultrasound parameters, specifically appendix diameter and presence of free fluids, did not significantly improve predictive performance across any of the evaluated models warrants careful interpretation within the constraints of the study design and available data. First, the retrospective nature of this analysis limits the standardization of ultrasound acquisition and interpretation, as examinations were performed by multiple operators with varying experience levels, potentially introducing measurement variability that could attenuate the true predictive signal of these imaging findings. Our finding that appendix diameter was a moderately influential predictor aligns with diagnostic studies that have refined the sonographic cut-off for appendicitis, with several authors advocating for a threshold of 7 mm in children to optimize diagnostic accuracy [27,28]. These observations support the value of standardizing ultrasound measurements as part of the initial risk stratification for pediatric appendicitis, even though its incremental contribution beyond clinical findings was modest in our prediction models. A more standardized imaging protocol, incorporating predefined scanning planes, consistent measurements of appendix diameter at a fixed anatomical location (such as the maximal outer-to-outer wall diameter in the transverse plane), systematic documentation of wall layer integrity, Doppler perfusion assessment, and uniform criteria for identifying free fluid or appendicolith, would have enhanced the consistency and reproducibility of these features across patients. Such standardization would reduce inter-operator variability, a well-recognized challenge in pediatric ultrasound, and could potentially unmask stronger associations between specific sonographic parameters and length of stay that were obscured in our retrospective dataset. Second, the ultrasound variables included in our models were limited to appendix diameter and free fluids, which, while commonly reported, represent only a subset of potentially informative sonographic features; other parameters such as appendiceal wall thickness, echogenicity, vascularity on Doppler assessment, and the presence of appendicolith or surrounding inflammatory changes were not consistently available or systematically captured in the dataset, and their inclusion might have yielded different results. Third, the relatively low prevalence of certain ultrasound findings in our uncomplicated appendicitis cohort (such as free fluids were present in only 46.4% of patients) may have reduced statistical power to detect small incremental effects. It is also possible that clinical parameters already encode much of the information captured by ultrasound, as inflammatory markers and physical examination findings reflect the underlying disease process that ultrasound visualizes, thereby limiting the added benefit of imaging once clinical data are available. Therefore, our results should not be extrapolated to complicated appendicitis or to settings where clinical examination is less reliable, and prospective studies with standardized ultrasound protocols and a broader range of imaging features are warranted to definitively establish the role of ultrasound in this prediction context.

We observed that the models struggled in predicting prolonged length of stay that can be attributed to several factors. First, the substantial class imbalance in our cohort is a critical factor, as only 7 patients (11.1% of the testing set) had prolonged stays, providing insufficient training examples for the models to learn the distinctive patterns associated with extended hospitalization. Second, prolonged length of stay in uncomplicated appendicitis may be driven by factors not captured in our preoperative predictor set, including postoperative complications such as wound infections or ileus, intercurrent illnesses, patient-specific psychosocial factors affecting discharge readiness, and institutional or logistical delays in arranging follow-up care or social support. Third, the pathophysiology of prolonged hospitalization may involve complex interactions between baseline clinical factors and unpredictable events during the hospital course, making it inherently more difficult to predict using admission data alone. Fourth, the relatively narrow range of length of stay in our cohort (mean 3.97 days, range 1–11 days) with most patients clustering around the mean may have limited the models’ ability to distinguish the tail end of the distribution. Future research should focus on oversampling or employing specialized algorithms for imbalanced regression, incorporating dynamic predictors that capture in-hospital events, and developing dedicated models specifically for identifying patients at risk of extended stays, potentially through multi-center collaborations that can provide larger cohorts of patients with prolonged hospitalization.

For hospital administrators and health policy makers, the implementation of such prediction models offers substantial opportunities for optimizing resource allocation, bed management, and operational efficiency in pediatric surgical units. Accurate prediction of length of stay can facilitate more effective scheduling of surgical procedures, improve patient flow, reduce unnecessary hospital days, and enhance the overall quality of care while containing costs. The integration of these models into routine clinical workflows could support value-based care initiatives by aligning clinical decision-making with efficient resource utilization, ultimately benefiting both patients and healthcare systems. Furthermore, the comparable performance between machine learning models and Bayesian approaches demonstrates that multiple analytical frameworks can be effectively employed for this prediction task, providing flexibility in implementation based on institutional preferences and available computational resources.

## 5. Strengths, Limitations and Future Directions

This study is the first comprehensive comparative evaluation of multiple ML algorithms alongside Bayesian methods, the integration of XAI to enhance clinical interpretability, and the rigorous sensitivity analysis along with bootstrap analyses confirming the robustness of findings across imputation strategies. However, several limitations warrant consideration, including the retrospective single-center design which may limit generalizability to other settings, the relatively modest sample size for certain subgroups particularly those with prolonged hospitalization, and the inherent challenges in capturing all clinically relevant predictors including post-operative recovery metrics and pain scores that may influence length of stay. Additionally, the substantial missingness in some urine parameters and the exclusion of the Pediatric Appendicitis Score to avoid multicollinearity may have impacted the comprehensiveness of the clinical models, while the lack of external validation in an independent cohort remains a critical gap that should be addressed in future work. Future research should focus on prospective multi-center validation of the developed models to confirm their generalizability across diverse populations and healthcare settings, while engaging clinicians in the co-design and implementation of these prediction tools to ensure seamless integration into existing clinical workflows. For researchers, opportunities exist to explore the integration of these models into electronic health records for real-time clinical decision support, investigate dynamic prediction approaches that update length of stay estimates as new clinical information becomes available during hospitalization, and evaluate the cost-effectiveness and clinical impact of these tools through prospective interventional studies. Collaborative efforts between clinicians and data scientists will be essential for translating these predictive models into actionable decision support systems that can meaningfully improve discharge planning, resource allocation, and ultimately patient outcomes in pediatric appendicitis care.

## 6. Conclusions

In conclusion, this exploratory study reveals that machine learning models, particularly Random Forest, predict length of hospital stay in uncomplicated pediatric appendicitis. The integration of XAI through SHAP analysis provided clinically interpretable insights, identifying the absence of peritonitis, appendix diameter, and inflammatory markers such as CRP and WBC count as the most influential predictors of hospitalization duration.

## Figures and Tables

**Table 1:** Baseline Characteristics Across Datasets.

Variable	Original Dataset (n = 323)	Imputed Dataset (n = 323)	Training Dataset (n = 260)	Testing Dataset (n = 63)	*p*-Value
Numerical Variables (Mean ± SD)					
Age (years)	11.33 ± 3.29	11.33 ± 3.29	11.43 ± 3.11	10.92 ± 3.95	1
Appendix diameter (mm)	8.50 ± 2.09	8.50 ± 2.06	8.61 ± 2.08	8.03 ± 1.91	0.94
BMI (kg/m^2^)	18.64 ± 3.99	18.60 ± 3.96	18.60 ± 4.02	18.63 ± 3.76	1
CRP level (mg/dL)	24.87 ± 40.28	24.87 ± 40.28	24.80 ± 41.09	25.19 ± 37.05	1
Hemoglobin (g/dL)	13.38 ± 1.16	13.38 ± 1.18	13.42 ± 1.18	13.25 ± 1.17	1
Length of stay (days)	3.97 ± 1.56	3.97 ± 1.56	3.93 ± 1.51	4.14 ± 1.75	1
Neutrophils (%)	74.45 ± 13.12	74.29 ± 13.34	74.15 ± 13.90	74.89 ± 10.83	1
RDW (%)	13.38 ± 5.88	13.36 ± 5.80	13.48 ± 6.44	12.83 ± 0.91	1
Temperature (°C)	37.40 ± 0.77	37.40 ± 0.77	37.39 ± 0.79	37.46 ± 0.72	1
Thrombocyte count (×10^3^/µL)	280.51 ± 59.13	281.17 ± 60.85	280.73 ± 60.37	282.98 ± 63.26	1
WBC (×10^3^ cells/mm^3^)	13.33 ± 4.77	13.33 ± 4.77	13.46 ± 4.85	12.79 ± 4.44	1
Categorical Variables [n (%)] ^a^					
Contralateral rebound tenderness	139 (43.4%)	139 (43.0%)	110 (42.3%)	29 (46.0%)	1
Coughing pain	103 (32.2%)	103 (31.9%)	91 (35.0%)	12 (19.0%)	0.58
Free fluids	143 (46.4%)	147 (45.5%)	115 (44.2%)	32 (50.8%)	1
Ipsilateral rebound tenderness	19 (7.5%)	27 (8.4%)	21 (8.1%)	6 (9.5%)	1
Ketones in urine	107 (45.0%)	147 (45.5%)	118 (45.4%)	29 (46.0%)	1
Loss of appetite	160 (49.8%)	161 (49.8%)	132 (50.8%)	29 (46.0%)	1
Lower right abdominal pain	315 (97.8%)	316 (97.8%)	256 (98.5%)	60 (95.2%)	1
Migratory pain	102 (31.8%)	102 (31.6%)	79 (30.4%)	23 (36.5%)	1
Nausea	191 (59.3%)	191 (59.1%)	156 (60.0%)	35 (55.6%)	1
Peritonitis—Generalized	14 (4.3%)	14 (4.3%)	12 (4.6%)	2 (3.2%)	1
Peritonitis—Local	103 (32.0%)	103 (31.9%)	77 (29.6%)	26 (41.3%)	
Psoas sign	89 (28.6%)	91 (28.2%)	69 (26.5%)	22 (34.9%)	1
RBC in urine	50 (21.1%)	62 (19.2%)	49 (18.8%)	13 (20.6%)	1
Sex: male	190 (58.8%)	190 (58.8%)	160 (61.5%)	30 (47.6%)	1
Sex: female	133 (41.2%)	133 (41.2%)	100 (38.5%)	33 (52.4%)	
Stool: abnormal	91 (28.5%)	93 (28.8%)	74 (28.5%)	19 (30.2%)	1
WBC in urine	33 (13.8%)	38 (11.8%)	31 (11.9%)	7 (11.1%)	1

Continuous variables are presented as Mean ± SD, and categorical variables were formatted as (%). ^a^—Percentages for the original dataset were estimated with the missing values excluded. BMI: Body mass index; CRP: C-reactive protein; RDW: Red blood cell distribution width; WBC: White blood corpuscles; and RBC: Red blood corpuscles.

**Table 2:** Performance Metrics of ML Models for Predicting Length of Hospital Stay.

Model	MAE	RMSE	MAPE (%)	P20 (%)
Random Forest				
Clinical	1.08 [0.80, 1.42]	1.66 [1.20, 2.15]	24.83 [19.21, 30.80]	58.73 [46.03, 69.84]
Clinical + US	1.10 [0.78, 1.46]	1.71 [1.19, 2.19]	24.67 [19.11, 32.06]	53.97 [39.68, 65.08]
XGBoost				
Clinical	1.30 [0.97, 1.71]	2.00 [1.46, 2.55]	31.64 [23.55, 41.75]	49.21 [36.51, 61.90]
Clinical + US	1.28 [0.93, 1.69]	1.97 [1.44, 2.53]	29.70 [22.04, 38.31]	44.44 [31.75, 57.14]

Data are presented as point estimates with 95% CI. MAE: Mean Absolute Error; RMSE: Root Mean Square Error; MAPE: Mean Absolute Percentage Error; P20: Percentage of predictions within 20% of actual values; US: Ultrasound.

**Table 3:** Performance Metrics of BART Models for Predicting Length of Hospital Stay.

Model	MAE	RMSE	MAPE (%)	P20 (%)
Clinical	1.07 [0.87, 1.50]	1.57 [1.21, 2.12]	25.10 [21.11, 35.00]	57.14 [30.16, 61.90]
Clinical + US	1.09 [0.88, 1.57]	1.62 [1.24, 2.21]	24.55 [21.31, 34.78]	53.97 [30.16, 61.90]

Data are presented as point estimates with 95% CI. MAE: Mean Absolute Error; RMSE: Root Mean Square Error; MAPE: Mean Absolute Percentage Error; P20: Percentage of predictions within 20% of actual values; US: Ultrasound.

**Table 4:** Exploratory Subgroup Analysis Performance Metrics for Random Forest Models.

Subgroup	n	RMSE	MAE	MAPE (%)	P20 (%)
Age Group					
<10 years ^a^	23	1.57 [0.74, 2.22]	1.03 [0.60, 1.54]	23.77 [15.97, 32.83]	52.17 [30.43, 73.91]
10–14 years ^a^	31	1.64 [0.90, 2.30]	1.02 [0.62, 1.49]	21.85 [15.37, 28.04]	64.52 [48.39, 80.65]
≥15 years ^a^	9	1.95 [0.66, 2.75]	1.45 [0.64, 2.29]	37.81 [16.27, 64.55]	55.56 [22.22, 88.89]
<10 years ^b^	23	1.57 [0.78, 2.28]	1.05 [0.65, 1.58]	23.75 [16.94, 31.19]	47.83 [30.43, 65.22]
10–14 years ^b^	31	1.70 [0.96, 2.41]	1.02 [0.61, 1.55]	21.10 [14.57, 28.16]	61.29 [41.94, 77.42]
≥15 years ^b^	9	2.08 [0.68, 3.05]	1.51 [0.62, 2.53]	39.33 [16.52, 68.16]	44.44 [11.11, 77.78]
BMI Category					
Underweight ^a^	39	1.56 [0.98, 2.09]	1.00 [0.66, 1.42]	22.07 [15.66, 28.93]	64.10 [48.72, 79.49]
Normal ^a^	20	1.72 [0.79, 2.51]	1.11 [0.64, 1.72]	23.99 [16.60, 31.48]	50.00 [30.00, 70.00]
Underweight ^b^	39	1.60 [0.99, 2.15]	1.02 [0.68, 1.44]	22.02 [16.42, 28.16]	61.54 [46.15, 76.92]
Normal ^b^	20	1.77 [0.79, 2.62]	1.10 [0.61, 1.77]	22.96 [15.27, 31.98]	45.00 [25.00, 65.00]
Sex					
Female ^a^	33	1.21 [0.67, 1.71]	0.79 [0.51, 1.14]	19.33 [13.87, 25.34]	75.76 [60.61, 90.91]
Male ^a^	30	2.05 [1.34, 2.77]	1.41 [0.95, 2.00]	30.88 [22.52, 42.64]	40.00 [23.33, 56.67]
Female ^b^	33	1.28 [0.68, 1.79]	0.80 [0.50, 1.16]	19.14 [13.45, 25.38]	66.67 [51.52, 81.82]
Male ^b^	30	2.09 [1.36, 2.76]	1.42 [0.93, 2.00]	30.75 [21.19, 41.54]	40.00 [23.33, 56.67]
Management					
Conservative ^a^	33	1.30 [0.74, 1.77]	0.87 [0.56, 1.20]	27.00 [18.18, 36.98]	60.61 [45.45, 78.79]
Primary Surgical ^a^	30	1.99 [1.21, 2.63]	1.32 [0.80, 1.86]	22.44 [16.24, 28.68]	56.67 [40.00, 73.33]
Conservative ^b^	33	1.32 [0.79, 1.82]	0.87 [0.57, 1.23]	26.61 [18.01, 37.44]	54.55 [36.36, 69.70]
Primary Surgical ^b^	30	2.06 [1.27, 2.71]	1.35 [0.82, 1.90]	22.53 [16.31, 29.06]	53.33 [33.33, 70.00]
LOS Category					
Short ^a^	31	1.17 [0.79, 1.59]	0.86 [0.60, 1.17]	30.08 [20.57, 40.96]	54.84 [38.71, 70.97]
Medium ^a^	25	0.71 [0.50, 0.90]	0.57 [0.41, 0.75]	12.36 [8.97, 15.98]	80.00 [64.00, 96.00]
Long ^a^	7	4.13 [3.25, 4.84]	3.92 [2.97, 4.78]	46.11 [37.70, 52.22]	Not estimable
Short ^b^	31	1.19 [0.76, 1.67]	0.81 [0.53, 1.13]	28.30 [18.51, 39.53]	51.61 [35.48, 67.74]
Medium ^b^	25	0.77 [0.58, 0.95]	0.63 [0.47, 0.81]	13.69 [10.33, 17.33]	72.00 [52.00, 88.00]
Long ^b^	7	4.25 [3.44, 4.97]	4.05 [3.14, 4.88]	47.77 [39.38, 54.42]	Not estimable

Data are presented as point estimates with 95% CI. RMSE: Root Mean Square Error; MAE: Mean Absolute Error; MAPE: Mean Absolute Percentage Error; P20: Percentage of predictions within 20% of actual values; LOS: Length of Stay. ^a^—Random Forest for Clinical model and ^b^—Random Forest for Clinical + US model.

## Data Availability

The data presented in this study are openly available in [UC Irvine Machine Learning Repository at [https://doi.org/10.5281/zenodo.7669442, accessed on 4 September 2026].

## Supplementary Materials

The following supporting information can be downloaded at: https://www.mdpi.com/article/10.3390/medsci14050556/s1, Table S1: Pattern of Missing Variables; Table S2: Comparison of SHAP Values between Random Forest Predicted Values within and outside P20.
