# Supplementary material for: Machine Learning-Predicted Length of Hospital Stay in Pediatric Appendicitis: An Exploratory Comparative Study of Clinical and Ultrasound-Based Models with Explainable Artificial Intelligence and Bayesian Analysis
Source: Med Sci (Basel). 2026 Sep 9;14(5):556. doi: 10.3390/medsci14050556 (PMC13608955; doi:10.3390/medsci14050556)
Supplement: Supplementary file 1 [file medsci-14-00556-s001.zip › medsci-4503594-supplementary.pdf]

Electronic Supplementary Table S1. Pattern of Missing Variables.

Variable	Missing count	Missing percent
RBC in Urine	86	26.63
Ketones in Urine	85	26.32
WBC in Urine	84	26.01
Ipsilateral Rebound Tenderness	71	21.98
Appendix diameter	31	9.6
Neutrophil count	28	8.67
Free Fluids	15	4.64
BMI	13	4.02
Psoas sign	12	3.72
RDW	9	2.79
Hemoglobin	5	1.55
Thrombocyte count	5	1.55
Stool	4	1.24
Contralateral Rebound Tenderness	3	0.93
Coughing Pain	3	0.93
Migratory Pain	2	0.62
Loss of appetite	2	0.62
Lower Right Abd Pain	1	0.31
Nausea	1	0.31
Temp	1	0.31
Peritonitis	1	0.31

Electronic Supplementary Table S2. Comparison of SHAP Values between Random Forest Predicted Values within and outside P20.

Clinical Model					
**Feature**	**Mean Absolute SHAP Success**	**Mean Absolute SHAP Failure**	**Fold Difference**	**P-Value**	**P-Adjusted**
Age	0.0497	0.0901	1.8114	0.0186	0.446156652
RBC_in_Urine_yes	0.0276	0.0143	0.5195	0.0375	0.900175497
BMI	0.0375	0.0425	1.1353	0.8953	1
Temp	0.0782	0.0771	0.9859	0.7870	1
WBC	0.0340	0.0598	1.7578	0.4412	1
Neutro	0.0523	0.0481	0.9196	0.3933	1
Hgb	0.0561	0.0423	0.7545	0.9393	1
Thromb	0.0474	0.0593	1.2507	0.8953	1
RDW	0.2242	0.1778	0.7932	0.1981	1
CRP_level	0.1095	0.1042	0.9513	0.3705	1
Sex_male	0.0091	0.0117	1.2815	0.0954	1
Migratory_Pain_yes	0.0074	0.0075	1.0223	0.8953	1
Contralateral_Rebound_Tenderness_yes	0.0328	0.0302	0.9213	0.5371	1
Coughing_Pain_yes	0.0048	0.0032	0.6748	0.4749	1
Nausea_yes	0.0030	0.0031	1.0102	0.9282	1
LOA_yes	0.0108	0.0151	1.3950	0.1201	1
Ketones_in_Urine_yes	0.0801	0.0875	1.0919	0.4249	1
WBC_in_Urine_yes	0.0010	0.0017	1.7394	0.8407	1
Stool_normal	0.0118	0.0124	1.0472	0.1981	1
Peritonitis_local	0.0697	0.0674	0.9666	0.9393	1
Peritonitis_no	0.3305	0.2826	0.8551	0.0847	1
Psoas_Sign_yes	0.0128	0.0118	0.9209	0.3344	1
Ipsilateral_Rebound_Tenderness_yes	0.0079	0.0151	1.9195	0.2632	1
Free_Fluids_yes	0.0421	0.0443	1.0512	0.5463	1

Clinical + US Model					
**Feature**	**Mean Absolute SHAP Success**	**Mean Absolute SHAP Failure**	**Fold Difference**	**P-Value**	**P-Adjusted**
Migratory_Pain_yes	0.0054	0.0038	0.7013	0.0252	0.630313587
Age	0.0526	0.0719	1.3674	0.7373	1
BMI	0.0446	0.0502	1.1255	0.6171	1
Temp	0.0686	0.0617	0.8994	0.5063	1
WBC	0.0401	0.0437	1.0894	0.6762	1
Neutro	0.0546	0.0463	0.8472	0.9182	1
Hgb	0.0280	0.0589	2.1032	0.9945	1
Thromb	0.0508	0.0878	1.7288	0.4467	1
RDW	0.2254	0.1888	0.8376	0.8534	1
CRP_level	0.0839	0.1029	1.2274	0.2751	1
App_Diam	0.2496	0.1738	0.6963	0.0913	1
Sex_male	0.0065	0.0073	1.1188	0.6075	1
Contralateral_Rebound_Tenderness_yes	0.0369	0.0359	0.9724	0.8749	1
Coughing_Pain_yes	0.0036	0.0033	0.8954	0.8426	1
Nausea_yes	0.0034	0.0046	1.3658	0.2293	1
LOA_yes	0.0094	0.0139	1.4863	0.0458	1
Ketones_in_Urine_yes	0.0681	0.0697	1.0224	0.5240	1
RBC_in_Urine_yes	0.0290	0.0249	0.8579	0.3990	1
WBC_in_Urine_yes	0.0020	0.0038	1.8732	0.1208	1
Stool_normal	0.0081	0.0086	1.0728	0.4146	1
Peritonitis_local	0.0382	0.0421	1.1026	0.4467	1
Peritonitis_no	0.3303	0.2938	0.8894	0.3617	1
Psoas_Sign_yes	0.0104	0.0112	1.0807	0.4633	1
Ipsilateral_Rebound_Tenderness_yes	0.0062	0.0131	2.1300	0.2851	1
Free_Fluids_yes	0.0346	0.0322	0.9310	0.2572	1
